# *Anisi Stellati Fructus*, a Significant Traditional Chinese Medicine (TCM) Herb and Its Bioactivity against Gastric Cancer

**DOI:** 10.1155/2022/4071489

**Published:** 2022-05-09

**Authors:** Maryam Khan, Saba Shamim

**Affiliations:** Institute of Molecular Biology and Biotechnology (IMBB), The University of Lahore, Defence Road Campus, Lahore, Pakistan

## Abstract

*Anisi stellati fructus* (ASF) is the fruit of *Illicium verum* Hook F. (Chinese star anise), which is native to many countries, and is a significant Chinese medicinal herb. Gastric cancer (GC) is one of the major fatal types of cancers with multiple stages and a poor prognosis. The present review aims to discuss the bioactive properties of ASF and its phytocompounds against GC, with a particular insight into the molecular mechanisms and signaling pathways involved in its anti-GC mechanism. Furthermore, it highlights the potential mechanism of action of major phytocompounds of ASF against GC. Clinical studies (*in vitro* and *in vivo*) regarding the action of ASF and its major bioactive compounds such as quercetin, luteolin, kaempferol, d-limonene, and honokiol against GC were reviewed. For this review, search of literature was performed in Science, PubMed, Google Scholar, Web of Science, and Scopus related to ASF and its phytocompounds, from which only relevant studies were chosen. Major bioactive compounds of ASF and their extracts have proven to be effective against GC due to the mechanistic action of these compounds involving signaling pathways that target cancer cell apoptosis, proliferation, and tumor metastasis in GC cells. Existing reports of these compounds and their combinatory effects with other modern anticancer agents have also been reviewed. From its traditional use to its role as an anticancer agent, ASF and its bioactive phytocompounds have been observed to be effective in modern research, specifically against GC. However, further studies are required for the identification of molecular targets and pharmacokinetic potential and for the formulation of anti-GC drugs.

## 1. Gastric Cancer (GC): Epidemiology and Etiology

Cancer is broadly defined as the uncontrolled proliferation of cells. It is classified into several stages before being termed fatal for a living system [1]. The World Health Organization (WHO) entails several factors to be involved in its progression, including lifestyle, diet, environment, gender, genetics, and overall health [2]. According to the Global Cancer Observatory (GCO), gastric cancer (GC) ranks the fifth most common cancer to be diagnosed globally and remains the third leading cause of cancer mortality after lung and colorectal cancer, respectively (5-year survival rate > 25%), with approximately 1 in 12 (<8%) cancer-related deaths being attributable to GC [3]. The mortality risk of GC from birth till the age of 70 is more than 1% and 0.5% for males and females, respectively [4]. According to the GLOBOCON project, there were more than 100,000 new cases of GC and more than 700,000 GC-related deaths in 2018. The occurrence of GC is variable with respect to region and ethnicity and is reported to be more prevalent (more than 2 times) in males than females in developed regions. In more than five countries of the world, GC has the highest prevalence among all types of cancers in males [5]. In countries of Central, Eastern Asia, and Africa, the prevalence rate of GC is the highest, whereas the lowest rate of incidence is in Korea, with almost 4 cases per 100,000 cases for males, respectively [6].

Though GC is one of the most fatal types of cancers, it is also one of the most influential types on the basis of human behaviors and therefore is preventable [7]. Till the 1980s, it was the leading cause of cancer-related deaths until it was overshadowed by lung cancer, as the rate of incidence of the latter was on the increase than the former, particularly in developed countries. Nevertheless, GC sustains a high mortality rate worldwide attributing to life year-burden [8]. There are a variety of factors that affect its development and manifestation, which can either be genetic or environmental and triggered by the presence or abundance of pathogens in the surroundings [9]. However, drastic changes in lifestyle, profound awareness, and pathogenic eradication have caused the incidental rate of GC to substantially decline over the years [10–12]. Nevertheless, GC is a progressive form of cancer, which is comprised of multiple stages, commencing from chronic superficial gastritis, atrophic gastritis, intestinal metaplasia, and dysplasia and adenocarcinoma [13]. Apart from these stages, gastric precancerous lesions (GPLs), including intestinal metaplasia and dysplasia, are significant indicatives of GC occurrence [14]. Various reports demonstrate the yearly prevalence of GC-related intestinal metaplasia, mild and moderate dysplasia, and severe dysplasia to be 0.25, 0.6, and 6% after a 5-year period of GC diagnosis [15]. Hereditary diffuse gastric cancer (HDGC) is one of the most common forms of GC, which is associated with familial history, caused by mutations in the cadherin 1 (*CDH1*) gene [16]. Other changes leading to the development of cancer that occur due to long-term inflammation include lack of balance between epithelial cell differentiation and apoptosis, atrophy and achlorhydria, and gastric colonization by enteric microorganisms with nitrate reductase movement, which encourages the development of cancer-causing nitrosamines. Corpus-dominating atrophy, or the deficiency of specific glandular cell types like parietal cells, is an important step towards the initiation of cancer [17].

## 2. Association of *Helicobacter pylori* with GC

The major risk factor for GC is the bacterium *Helicobacter pylori*, which was discovered by Barry Marshall and Robin Warren in 1982, prior to which other factors such as lifestyle, diet, and stress were considered to be major risk factors for gastric disease [18]. *H. pylori* is a Gram-negative, spiral-shaped bacterium, which predominantly resides in the human stomach and is reported to colonize the human gut of immunocompromised individuals during infection [19]. It is a stomach pathogen that is responsible for stomach-related ulcers (duodenal and gastric) and cancer (gastric, mucosa-related lymphoid tissue lymphoma) in infected hosts [20]. The incidence of *H. pylori* has been reported to elevate the risk of stomach cancer to a fivefold ratio within a decade of infection. Moreover, more than 90% of noncardia subtypes are reported to be associated with the pathogen [21]. Polymorphisms of IL-10 and IL-17, which are associated with GC, are also involved in the interaction with *H. pylori* infection [22]. Histopathologically persistent gastritis, gastric decay, intestinal metaplasia, dysplasia, and malignancy are different phases which ultimately occur due to *H. pylori* infection, more than often leading to mortality [23]. The role of *H. pylori* in the initiation of GC involves direct infection and inflammation that occurs in the gastrointestinal mucosa of the host [24]. For instance, genes for the Type IV secretion system, which are reported to be essential for the transport of CagA proteins into human epithelial cells by *H. pylori*, were observed to be abundant in the intragastric microbiome of intestinal metaplasia patients [25]. Cag-Pathogenicity Island (cagPAI) consists of more than 30 genes that encode the Type IV secretion system and the CagA protein. Strains of *H. pylori* that express cagPAI are associated with pathogenesis of gastric disease, peptic ulcers, and gastric cancer [26]. CagL, another protein, aids in translocating CagA and secreting IL-8 from *H. pylori*. *In vitro* studies have reported the role of CagA in inducing tumorigenesis in AGS cells [27]. Abl and Src kinases phosphorylate CagA on tyrosine residue at 4 distinctive EPIYA motifs inside the host cell, which results in several morphological cellular changes and increased cellular migration [28]. These EPIYA motifs are significant as their phosphorylation status, and the amount is an indicator for GC [29]. Tyrosine-phosphorylated CagA activates tyrosine phosphatase in the host cell, which in turn activates ERK1/2 and C-terminal Src kinase, while the interaction between the two results in cell elongation [30]. Even in its nonphosphorylated form, CagA has several pathogenic effects by targeting various cellular components like E-cadherin, c-Met, and Grb-2 to mediate proinflammatory responses, disrupting cell to cell apical junctions, activating *β*-catenin, and resulting in the loss of cell polarity [31, 32].

The highly pathogenic cytotoxin secreted by *H. pylori*, vacuolating cytotoxin A (VacA), is associated with various genotypes and vacuolating activities. Patients infected with *H. pylori* strains that express VacA with s1 or m1 genotype are reported to have an elevated risk of gastric cancer. Therefore, VacA expression has been designated as a significant biomarker for the development of gastric cancer [33]. For the evasion of the bacterium from the host's immune system, genetic diversity is one of the major factors that *H. pylori* undertakes for the initiation of chronic inflammation and host colonization. Previous literature reported the involvement of *CagA* and *VacA* genes in the progression of neoplastic or nonneoplastic GC [34]. Moreover, *H. pylori* produces the enzyme urease, which releases ammonium and carbon dioxide for the neutralization of stomach acids, allowing the survival of the bacterium. Ammonia further causes alterations in the tissue structure, whereas carbon dioxide offers protection to *H. pylori* from host immune cells and induces angiogenesis, thus promoting GC [35]. Apart from GC, disturbance in the tissue microenvironment (TME) of the host gastric mucosa [36, 37] also involves *H. Pylori* infection. Furthermore, other bacterial species than *H. pylori* can also be attributable to carcinogenesis in the gastric mucosa [38].

Apart from bacterial pathogens, the occurrence of GC can be attributable to the infection caused by viruses, including Epstein-Barr virus (EBV), which has been reported to be associated with more than 9% of GC cases [39]. Its role in the manifestation and growth of GC has been regarded as a complex one, as many other factors are commonly associated with the incidence of this disease [40]. Like *H. pylori*, EBV's role in GC is also linked with genetic mutation, characterized here by the posttranscriptional genetic regulation of EBV by miRNAs [41].

## 3. Treatment of GC: Conventional and Modern Methods

### 3.1. Conventional Methods and Chemotherapeutic Agents

Chemotherapy, radiotherapy, tumor resecting surgery, immunotherapy, and targeted therapy have all proven to be effective against GC and adenocarcinoma, which implies that a multidisciplinary approach is pertinent for the suitable selection of treatment. Chemotherapy for resectable GC is now acceptable, but classification of GC on the grounds of various molecular subtypes is significant for a more personalized, therapeutic approach. Random clinical trials prove evidence that perioperative and postoperative chemotherapy, chemoradiation, and immunotherapy are also considerable options for treatment [42]. Several cytotoxic agents are reported to be active in stages of advanced GC, including irinotecan, platinum, and taxanes. Oxaliplatin is the preferred platinum in most treatment regimens [43]. In the second-line of treatment for metastasized GC, monoclonal antibodies such as ramucirumab have proven a boost in survival of GC patients, with both its singular therapy and use in combination with paclitaxel being deemed effective. Kinase inhibitors such as lenvatinib and regorafenib have also been investigated in their use in immunotherapy in GC in East-Asian populations [44]. Nevertheless, the choice and options available for treatment are dependent on the prognosis of disease and the response of the patient to the preferred method, as these treatments come with their set of side effects.

### 3.2. Traditional Chinese Medicine (TCM) and GC

Over the past years, significant research in the field of cancer has resulted in scientific breakthroughs, including cancer immunotherapy, whose efficacy is dependent on the inflammation regulating the tumor microenvironment [45]. However, this approach is restricted to a slow rate of response, thus making it appropriate only for some patients [46]. This rate can be accelerated by the combination of immunotherapy with other agents, which can lead to an increase in the cure rate [47]. Traditional Chinese medicine (TCM) is a conjugative discipline, combining personalized medicine with therapeutics and cancer therapy. However, a greater part of the patients presently uses TCM as an alternate method of pain alleviation rather than the main method of treatment, despite the fact that it has been directly associated with treating major diseases such as cancer in recorded medical history [48].

Conventional treatments for diagnosed GC include tumor resection during early stages, but unfortunately, in patients with advanced stages of nonresectable tumor, the patients are advised noninvasive herbal therapy regimens meant to only alleviate their pain and increase their life expectancy [49]. Since time immemorial, plants have been used as therapeutic sources for the treatment of infections, diseases, and healthcare. Decades worth of studies have proven their efficacious potential and discovery of medicinal plant-derived drugs. The Chinese Pharmacopeia deciphers the involvement of Chinese herbs, plants, and their concoctions in addressing, managing, and curing clinical and terminal diseases. TCM includes medicinal plants and their associated constituents that can be used for therapeutic as well as theranostic purposes. They provide a wide spectrum of information about the mode of action of medicinal plants regarding managing a variety of diseases and simultaneous information about the human physiological systems [50]. Earliest reports of its use date back to 200 AD for the improvement of healthcare. Currently, traditional medicinal documentation of Ayurvedic and Chinese medicinal plants is available online in various databases. They contain comprehensive information about different plant parts, secondary metabolites, and bioactive compounds. In developing countries, people still employ traditional concoctions, which have been passed on from generations to cure diseases. This practice is now being opted by even those who reside in developed countries, where innovative research is being conducted to unlock the bioactive mechanisms effective against various diseases such as cancer [51]. Bioactive phytoconstituents that address cancer cells include alkaloids, saponins, terpenoids, tannins, and flavonoids [52]. In a similar manner, TCM has proven to be effectual in the prevention of GPL progression [53].

Traditional medicine is comprised of the comprehension of beliefs, knowledge, and information used in amalgamation with disparate therapies and other practices, which gives way to the incorporation of herbal medicines prepared from animals, minerals, and/or plant and its constituents for diagnosis, treatment, and prevention of infection and disease [54]. Therefore, in context, every geographical locale inherits its own rich knowledge of traditional medicine, which may be passed onto generations and spanning decades. In developing and developed countries, this built-up indigenous knowledge lives on to serve fundamental roles in employing localized resources like plants and herbs [55]. Therefore, this review sheds light on one of these herbs, *Anisi stellati fructus*, for its mechanism of action and treatment of GC.

## 4. An Example of TCM: ASF and Its Activity against GC

*Anisi stellati fructus* (ASF) is the star-shaped fruit of *Illicium verum* Hook F. (Chinese star anise), which belongs to the Illiciaceae family [56], but the former is reported to be a member of the Schisandraceae family [57]. *I. verum* is a highly regarded Chinese medicinal herb which is also mentioned in the Chinese Pharmacopeia [58]. There are various species in the genus, which demonstrate variance due to their distinctive morphology, composition, and growth habitat. *I. verum* is an aromatic, medium-sized tree which is grown in areas native to Jamaica, Laos, Japan, Indonesia, Philippines, and north-east and south-west Vietnam and China, respectively [56, 59], and is widely distributed in many regions of Asia and North America [60]. In various regions all over the world, it goes by different local names, including “Bādiyān” (Persian), “Badiyaan” and “Badiyaan ka phool” (Urdu), “Phoolchakri” (Hindi), “Badiane” (French), and star anise (English). In TCM, it is commonly known as Ba Jiao Hui Xiang [58, 61].

### 4.1. ASF in Culinary Practice

ASF is characterized by 6–8 ridged follicles that are star-shaped, woody, and wrinkled in texture. It is generally regarded as safe and nontoxic for consumption [62] and has traditionally been used as a staple spice in various cuisines, including Oriental, Indian, and Pakistani cuisines. It is a major component of the widely used five-spice powder (locally known as garam masala in the Indian subcontinent) used in the preparation of stews and curries [63]. In European countries, it is used in the preparation of alcoholic drinks along with various teas, fruit jams, and condiments [64].

### 4.2. Medicinal Uses of ASF

The use of ASF in treating various infections and diseases is practiced in various regions, including Asia and North America [65]. It is reported to possess antimicrobial, antiviral, and antioxidant properties [66]. In Chinese, Ayurvedic, and Unani medicine, it is reported to improve digestion and alleviate symptoms of dysentery, dyspepsia, asthma, flatulence, menstruation irregularities, colic, inflammation, bronchitis, and rheumatic diseases. Using it in a concoction for herbal teas can relieve cough and flu and can reinvigorate various organs of the human body [61].

### 4.3. Anticancer Properties of ASF

Though the effects of ASF and its extracts and decoctions against cancer and tumor growth have not been thoroughly established, few studies have brought them to light, providing a brief yet informative insight into its anticancer mechanism. Extracts of ASF inhibited angiogenesis in Human umbilical vein endothelial cells (HUVECs) at concentrations of 10 *μ*g/ml, suggesting its anticancer activity [67]. Another study reported that the oral administration of ASF resulted in the decrease of metastasis in lung cancer cells with little or no cytotoxic effects [68]. In chronic myeloid leukemia (CML) cells, ASF and its combinational treatment with imatinib yielded antileukemic activity, indicating that ASF could be considered as a potential agent for CML therapy [69]. Therefore, these studies suggest that ASF possesses significant anticancer activity, which could be further elucidated by similar findings.

### 4.4. Chemical Constituents of ASF

The fruit of *I. verum* is reported to contain various alkaloids, essential oils, and tannins, with significant amounts of cis- and trans-anethole, limonene, safrole, *α*- and *β*-pinene, *β*-phellandrene, *α*-terpineol, and farnesol [70]. Flavonoids like quercetin and kaempferol and their glucosides, phenolic compounds like shikimic acid, and fatty acids such as linoleic, myristic, stearic, betunolic, and phenyl propionic acid are also reported to be active constituents of ASF [48, 71]. Furthermore, the essential oils of ASF are comprised of flavonoids, terpenes, sesquiterpenes, and lignans which are equally significant respective to their medicinal and therapeutic properties. Additional bioactive compounds which are found in ASF essential oils are myrcene, limone, linalool, luteolin, estragole, caryophyllene, *γ*-terpineol, and *α*-humulene [72]. Moreover, recent studies have reported the presence of other compounds such as *β*-sitosterol, *α*-phellandrene, *β*-myrcene, mairin, honokiol, cineol, and safrole [73–75].

## 5. Major Bioactive Compounds of ASF and Their anti-GC Effects

ASF and its compounds have been extensively reported for their anticancer activity [76, 77]. Kim et al. reported that the oral administration of ASF significantly decreased the metastasis in malignant cancer cells, which ultimately resulted in the reduction of MMP-9, MMP-13, MMP-14, uPA, and gelatinase activities by its treatment [68]. It also inhibited the activation and phosphorylation of NF-*κ*B, AP-1, and p38 pathways, respectively, as well as suppressing tumor angiogenesis in cells. Another recent study examined the effect of ASF extract on CML cells, which demonstrated that the treatment induced cytotoxicity and proliferation inhibition in a dose-dependent manner. The combination of ASF extract with imatinib (IM) also leads to an apoptotic effect in the cells, which was not as pronounced as the singular treatment of IM on CML cells [69]. In a similar manner, many bioactive compounds of ASF (present in a major or minor amount) have been identified to be effective against GC, which are mentioned in detail in the next section.

### 5.1. Quercetin

**Figure 1:**
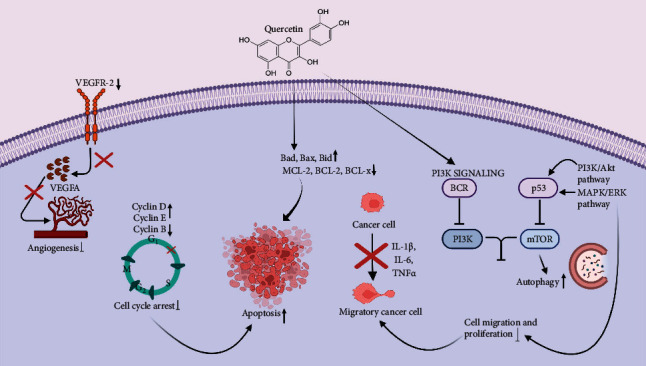
Anticancer effect of quercetin against GC.

Quercetin (3,3′,4′,5,7-pentahydroxyavone) is a flavonoid which is abundantly found in many foods and plants, with many properties attributed to the compound, including antioxidative, anti-inflammatory, antimicrobial, and anticancer activities, respectively [78, 79]. The treatment of quercetin in cancer cells has been reported to induce apoptotic, antiulcer, and chemopreventive effects [80]. Furthermore, quercetin facilitates the prevention of mucosal damage in gastric ulcer formation, which is also said to be associated with its antibacterial action against *H. pylori* [81]. Various studies have reported the effectiveness of quercetin against cancer proliferation and angiogenesis. Its effect on gastric cancer apoptosis was revealed through Bax, BCL-2, and caspase analyses [82]. The regulation of P450 enzyme expression also resulted in the activation of procarcinogens by quercetin. Its treatment has also been affiliated with the enhancement of DNA repair and the elimination of carcinogens and actively proliferating cancer cells [83]. Quercetin also mediates apoptosis by the expression of various proteins such as mitogen-activated protein kinases (MAPK), phosphatidylinositide 3-kinases (PI3K), and protein kinase C (PKC) through regulating the expression of the BCL-2 family [84]. In two studies, it was demonstrated that quercetin and its combinative treatment with other cancer-inhibiting agents induce autophagy in GC cells *via* the negative regulation of the Akt-mTOR signaling pathway, which leads to the overall inhibition of cellular proliferation [85]. The administration of quercetin also leads to the reduction in the invasion and migration of GC cells through downregulating the expression of uPAR and uPA proteins, respectively [86]. In GC cell lines, the treatment of quercetin is demonstrated to reduce the progression of cellular growth in the cell cycle phases [87]. The combinative treatment of quercetin with curcumin resulted in the decrease of Akt and ERK phosphorylation, which suggested cellular apoptosis *via* the mitochondrial pathway in GC cells [88]. In *in vivo* models, the treatment of quercetin stimulated the generation of nitric oxide synthase (nNOS), which reacted with ROS for the inhibition of cellular proliferation of cells of the gastric mucosa, which were previously treated with ethanol [89]. Other *in vivo* studies depicted the administration of oral quercetin to reduce COX-2, Twist1, and ITG*β*6 levels [90]. Furthermore, the downregulation of angiogenesis-related factors (VEGFA and VEGFR-2) also suggested the effectivity of quercetin against GC cells [91]. In the AGS cell line, quercetin leads to the reduction and increase in the expression of antiapoptosis (MCL-2, BCL-2, and BCL-x) and proapoptosis (Bad, Bax, and Bid) related proteins, respectively [92]. The blocking of the phosphoinositide 3-kinase- (PI3K-) Akt pathway is one of the mechanisms through which quercetin is reported to inhibit the mitochondrial pathways leading to the progression of GC, which was reported in many studies (Figure 1) [84, 93]. Recent *in silico* analyses have employed the network pharmacology approach for understanding the mechanism of quercetin and its involvement in molecular pathways against GC [94, 95].

### 5.2. Luteolin

Luteolin (3′,4′,5,7-tetrahydroxyflavone) is another well-known flavonoid that has been widely reported to be effective against the progression of several types of cancers, including GC [96]. In a study, luteolin was reported to inhibit the proliferation of GC cells through suppressing the Notch signaling pathway [97]. The administration of luteolin leads to the reduction in cell viability and induction of cell cycle arrest, as well as apoptosis in GC cell lines [98]. The treatment of GC cell lines with luteolin alone induced apoptosis, while its combinatory treatment together with cisplatin resulted in the down and upregulation of CDC2, CDC25C, Cyclin-B1, and p21/cip1, respectively, leading to the effective inhibition of cell growth [96]. Lu et al. reported that luteolin was attributable to the downregulation of c-Met, MMP9, and Ki-67 in GC cells while promoting the induction of apoptosis *via* activating apoptotic proteins (CAS3 and PARP1), thus suggesting that luteolin can target Akt/ERK signaling pathway for its anti-GC effect [97].

**Figure 2:**
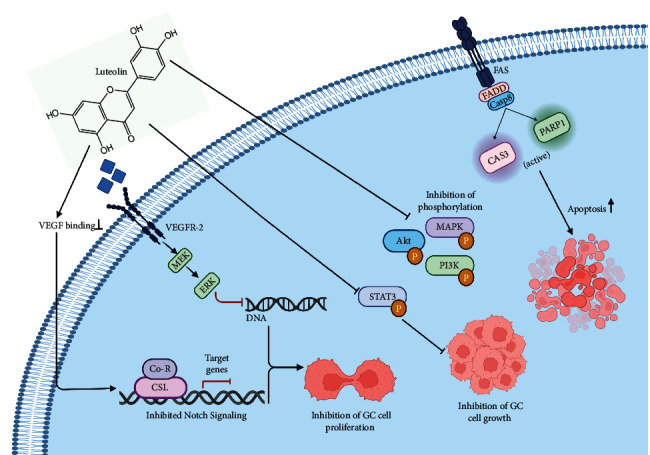
Anticancer mechanisms of luteolin against GC.

BCL-2 is an apoptosis regulating protein that is characteristically found to be overexpressed in various cancers [98]. In a study, new findings demonstrated luteolin to downregulate the expression of the protein *via* the upregulation of miR-34a, thus aiding in inhibiting cellular growth in GC [99]. The suppression of phosphorylation of MAPK, AKT, and PI3K signaling pathway, as well as the induction of apoptosis in GC cells, was also observed through the regulation of CAS3, CAS9, and Bax/BCL-2 ratio by luteolin [100]. It is reported that treating GC cells with luteolin leads to the inhibition of STAT3 phosphorylation, reducing the growth of tumors *in vivo* [101]. Notch signaling pathway is reported to be associated with cellular angiogenesis and the regulation of AKT, MMP-9, and NF-*κ*B signaling pathways [102], the latter of which in turn regulates VEGF expression in various human tissues [103]. In GC, luteolin impedes the expression of VEGF *via* the downregulation of the Notch1 pathway, the study findings of which have been proven previously by two studies [97, 104]. Additionally, treating *H. pylori*-infected GC cells with luteolin resulted in the induction of IL-8 and NF-*κ*B at both protein and mRNA levels, respectively [105]. Furthermore, two recent studies have investigated the combinatory synergistic effect of luteolin with oxaliplatin in GC cells. The combined treatment demonstrated the positive effect of both agents on the inhibition of cellular proliferation, activation of CytC/caspase signaling, and the induction of cell cycle arrest (GC/M phase) and cellular apoptosis [106] (Figure 2).

### 5.3. Kaempferol

**Figure 3:**
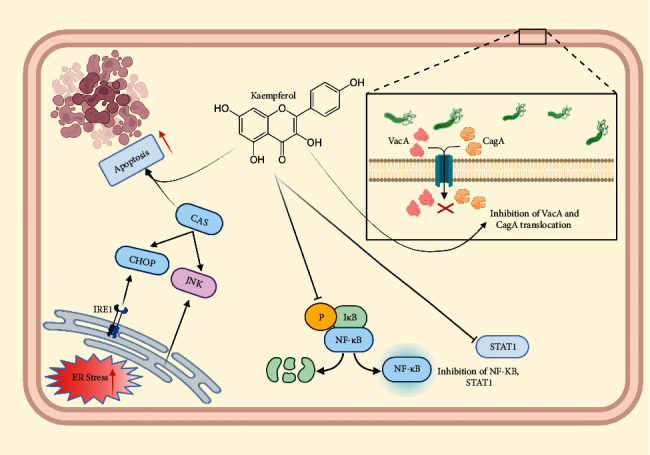
Mode of action of kaempferol against GC.

Kaempferol is a flavonoid compound that is abundantly found in various plants (edible and medicinal). Its various biological activities also include the anti-inflammatory property, which is apparently useful in inhibiting the expression of several proinflammatory cytokines, NF-*κ*B, STAT1, and AP-1 [107, 108]. Therefore, kaempferol has been investigated and reported to be effective in many types of cancers, including GC [109, 110]. In a study conducted in Spain, kaempferol consumption reduced the risk of GC, while an *in vivo* study reported the inhibition of cancerous cell growth in GC xenografts, thereby suggesting the antiproliferative and metastasis-inhibiting ability of the flavonoid [111, 112]. Li et al. examined the effect of kaempferol against injury to the gastric mucosa, which was induced by ethanol [113]. The treatment with kaempferol demonstrated its protective effect by facilitating the inhibition of MPO and proinflammatory cytokine levels, as well as improving NO production in cells. In AGS cells, kaempferol leads to a decrease in the expression of IL8, IL-1*β*, and TNF-*α*. Moreover, its anti-inflammatory effect was observed by the suppression of the translocation of CagA and VacA proteins of *H. pylori* in GC cells [114]. Kaempferol is also reported to induce autophagy and apoptosis in GC cells *via* activating the IRE1-JNK-CHOP signaling and AMPK*α*/ULK1 pathway and by positively regulating ER stress in GC cells [111, 115] (Figure 3). A recent study also shed light on the mode of action of the compound against GC through a network pharmacology approach [116]. Shrestha et al. reported the reduction in G9a expression, as well as inhibition of mTOR signaling and cellular proliferation in GC cells after kaempferol treatment [117].

### 5.4. Honokiol

Honokiol (3,5-di-(2-propenyl)-1,1-biphenyl-2,2-diol) is a small, biphenolic lignan. It has been reported to suppress Akt and NF-*κ*B activation, which in turn results in the phosphorylation and degradation of I*κ*B*α*, respectively [118]. It is also reported to mediate the suppression of STAT3 activity previously induced by IL-6, where the activated form of the former has been associated with many cancer cells [119]. The treatment with honokiol has been reported to be effective against GC, where it was attributable to the induction of apoptosis and downregulation of COX-2 and PPAR-*γ* in GC cells [120]. The significant anticancer activity was observed to be correlated with GRP94 levels, which were found to be reduced after administration of honokiol in mice in a dose-dependent manner [121]. Therefore, it can potentially serve as a promising anticancer therapeutic target for several pathways [122]. Liu et al. observed that honokiol increases SHP-1 activity that subsequently leads to the deactivation of the STAT3 pathway, thereby suggesting that honokiol actively inhibits cellular angiogenesis and proliferation of GC cells [123]. In a study, treatment with honokiol revoked the down- and upregulation of E-cadherin and TPL2, respectively, the latter of which was observed to be subsequently associated with decreased growth and vascular density *in vivo* [124]. This mechanism of action, combined with the inhibition of epithelial-to-mesenchymal transition as well as regulation of apoptosis (induced by ER stress), serves a key role in the therapeutic action of honokiol against GC. In a recent study, the anticancer properties of honokiol were attributable to its ability to downregulate PPAR-*γ* activity, as well as the expressions of CDC25C, CDC2, and Cyclin B1, which aid in inducing ER stress which in turn decreases vascular density [125].

### 5.5. D-Limonene

D-Limonene is a monoterpene compound that has been reported to possess anticancer activities against different cancers [126, 127]. Lu et al. evaluated that d-limonene can inhibit the proliferation of GC cancer cells *via* the induction of apoptosis in cancer cells [128]. It has also been attributable to strong antioxidant activity resulting in the inhibition of H_2_O_2_-induced CAS3, CAS9, and p38/MAPK activation, as well as a decline in the BCL-2/Bax ratio, thereby indicating that it could offer protection against oxidative stress [129]. Another study evaluated that oral administration of limonene (≥400 mg/kg) in mice led to a reduction in tumor mass weight [128]. The combination of d-limonene with berberine and its singular treatment on GC cell line resulted in the increased expression of CAS3 and ROS, reduced expression of BCL-2, and cell cycle arrest, indicating that d-limonene causes apoptosis *via* the regulation of the mitochondrial pathway [130–132].

## 6. Bioactivity of ASF Compounds against *H. pylori*

*Helicobacter pylori* is often recognized to have a strong correlation with the occurrence of gastric diseases, including GC. Many bioactive compounds have been associated with the anti-*H. pylori* activity and other protective effects, which subsequently promote good gastric health and protection from several diseases. In a study, luteolin was observed to inhibit the activity of the arylamine N-acetyltransferase (NAT) enzyme, which is responsible for the N-acetylation of PABA and AF in *H. pylori* [133]. Another study demonstrated the protective effect of quercetin against *H. pylori* in the corpus mucosa [134]. Though the main mechanism of action of quercetin is not clear, it is reported to be associated with the declining activity of urease, as well as its ability to chelate iron, which is a major cofactor imperative for *H. pylori* growth. Treatment of quercetin in *H. pylori*-infected animals is reported to reduce the process of inflammation and bacterial count in the gastric mucosa [135].

*β*-Caryophyllene is a naturally occurring bicyclic sesquiterpene that is widely found in many medicinal plants and is reported to possess many protective abilities, including antibacterial and anti-inflammatory properties. In a study, *β*-caryophyllene demonstrated significant gastroprotective activity in an ethanol-induced gastric ulcer model, reducing the lesions by more than 70% [136]. A recent study by Shim et al. revealed that the treatment of *H. pylori*-caused gastrointestinal disease with *β*-caryophyllene demonstrated remarkable improvement in physiological symptoms and subsequently resulted in IL-1*β* decrease in serum [137]. Furthermore, the anti-*H. pylori* action of similar compounds has also been well-reported in many studies [114, 138, 139].

## 7. Future Perspectives and Conclusions

Medicinal herbs such as ASF have been traditionally used for the treatment of several ailments. These herbs are deemed indispensable as therapeutic candidates for treating various diseases such as cancer. The protective effects of ASF and its compounds against GC are majorly related to modulating major hallmarks of cancer, such as the inhibition of cellular proliferation, angiogenesis, inducing apoptosis as well as the suppression of cell migration, and the promotion of immune cell secretion against GC cells (Table 1). Furthermore, the ability to use ASF and its compounds in combination with other anticancer agents and/or as adjuvants in cancer immunotherapy is also an exciting and encouraging field of study, as results have been promising in recent investigations. These properties could provoke great economic interest for their pharmaceutical applications when the compounds are extracted, as compared to their laboratory synthesis. In this regard, future studies and phytochemical analyses of ASF and its bioactive compounds may result in the discovery of novel anti-GC targets and may also lead to improvements in their chemical synthesis. This approach has been further elucidated in studies of single, isolated compounds, such as quercetin, where its standalone efficacy against GC is observed to be far greater than the herb on the whole. Therefore, these phytocompounds pose a superior anti-GC potential, which could be elucidated in further *in vitro* and *in vivo* studies and clinical trials.

## Figures and Tables

**Table 1:** The anticancer effect of various bioactive compounds of ASF against gastric cancer *in vitro*.

Compound	Cell line	Concentration used	Effect on protein/pathway (s)	References
Quercetin	AGS and MKN28	10–160 *μ*M	Inhibit Akt-mTOR pathway	[85]
	AGS	Quercetin alone (6.25, 12.5, 25, 50, and 100 *μ*M) With SN-38 (5-25 nM)	Downregulate VEGFA and VEGFR-2 ↓ COX-2, Twist1, and ITG*β*6	[91]
	SNU719 and MKN74	N/A	Inhibit EBNA-1 and LMP-2 proteins ↑ Cleaved CAS3, CAS9, and PARP Induce p53, Bax, and Puma	[90]
	BGC-823	5, 30, 60, 90, and 120 *μ*mol/L	Induce CAS3, Bcl-2, and Bax ↓ Bcl-2/Bax ratio ↑ CAS3 expression	[82]
	BGC823 and AGS	10 *μ*M	↓ Cell migration and invasion ↓ uPA and uPAR expression ↓ MMP2 and MMP9 activity inhibit Pak1-Limk1-cofilin, NF-*κ*B, PKC-*δ*, and ERK1/2 signaling, and AMPK*α* activation	[86]
	GCSC	20–100 *μ*M	Inhibit (PI3K)-Akt signaling	[84]
	HGC-27, NUGC-2, MKN-7, and MKN-28	70 *μ*M (IC_50_-32–55 *μ*M)	Cell cycle arrest (Gi to S phase)	[87]

Luteolin	AGS	50 *μ*M (24 h) 80 *μ*M (48 and 72 h) IC_50_ 29.6 ± 3.8 (48 h) and 23.5 ± 2.4 *μ*M (72 h)	↓ CDC2, cyclin B1, and CDC25C levels ↑ Apoptosis, CAS3, CAS6, CAS9, Bax, and p53 ↓ BCL-2	[96]
	CRL-1739	30 *μ*M	Induce IL-8 expression ↑ NF-*κ*B mRNA expression	[105]
	MKN45 and SGC7901	20 *μ*M (24 h) 40 *μ*M (48 h) 80 *μ*M (72 h)	↑ Cleaved CAS3 and PARP; induce apoptosis Downregulate MMP9 expression and c-Met/Akt/ERK signaling	[97]
	MKN45 and BGC823	40 *μ*M	↑ Apoptosis Inhibit GC cell proliferation, cyclin D1, cyclin E, BCL-2, MMP2, MMP9, N-cadherin, and vimentin Induce p21, Bax, E-cadherin expression, Notch1, PI3K, AKT, mTOR, ERK, STAT3, and p38 signaling pathway	[98]
	BGC-823	0–60 *μ*M (48 h)	↑ Cleaved CAS9 and CAS3 ↓ p-PI3K, p-AKT and p-mTOR, and p-ERK1/2	[100]
	MFC	Luteolin alone (20 *μ*M) and/or oxaliplatin (5 *μ*M (24 h))	Downregulate ERK1/2 phosphorylation and activation Combined treatment induced cell cycle arrest (G2/M phase) Induce apoptosis	[106]
	SGC-7901	40 *μ*M (24 h)	Combined treatment inhibited proliferation Induce ERK1/2 phosphorylation, JNK, and P38 MAPK signal transduction Inhibit PI3K/AKT and ERK1/2 MAPK intracellular signaling Induce apoptosis	[99]
	MKN28, SGC7901, and GSE-1	60 *μ*M	Cell cycle arrest (G2/M phase) ↓ Cyclin B1, CDK1 and CDC25C, COX-2, p-AKT, and p-ERK ↑ Cleaved CAS3, CAS9, PARP	[101]

Kaempferol	AGS, SNU-216, NCI–N87, SNU-638, and MKN-74	25 *μ*M, 50 *μ*M, and 100 *μ*M (24 h)	Activate IRE1-JNK-CHOP signaling pathway Induce apoptosis LC3-I to LC3-II conversion Downregulate p62	[112]
	Rh30	25 or 50 *μ*M kaempferol and quercetin (24 h)	Induce apoptotic markers (cleaved PARP and CAS3) Inhibit cell growth, survival, migration, and invasion by blocking mTOR signaling	[117]

D-Limonene	HLEC	125–1800 *μ*M	↓ H_2_O_2_-induced ROS generation and BCL-2/Bax ratio Inhibit CAS3, CAS9 activation, and p38 MAPK phosphorylation	[129]
	MGC803	80 *μ*M (24–48 h)	↓ Mitochondrial transmembrane potential (DCm) ↑ CAS3 expression ↓ BCL-2 expression	[131]

Honokiol	AGS, MKN45, N87, and SCM-1	20 and 50 mM	Induce apoptosis Activate 15-LOX-1 expression Regulate PPAR-g and COX-2 pathway	[120]
	AGS, MKN45, and SCM-1	20 mM IC50–AGS (20 mM) and MKN45 and SCM-1 (40 mM)	Induce SHP-1 activity and STAT-3 dephosphorylation Activate ER stress and calpain-II regulation	[123]
	AGS and MKN45	20 *μ*M	Inhibit TGF*β*1- or MNNG-induced EMT	[124]
	AGS, N87, MKN45, and SCM-1	5–40 *μ*M	↓ Glucose-regulated protein (GRP94)	[121]

## Data Availability

No data were used to support this study.
